# Hasse diagram as a green analytical metrics tool: ranking of methods for benzo[*a*]pyrene determination in sediments

**DOI:** 10.1007/s00216-016-9473-4

**Published:** 2016-04-01

**Authors:** Paulina Bigus, Stefan Tsakovski, Vasil Simeonov, Jacek Namieśnik, Marek Tobiszewski

**Affiliations:** Department of Analytical Chemistry, Chemical Faculty, Gdańsk University of Technology (GUT), 11/12 G. Narutowicza St., 80-233 Gdańsk, Poland; Chair of Analytical Chemistry, Faculty of Pharmacy and Chemistry, University of Sofia “St. Kl. Okhridski”, J. Bourchier Blvd. 1, 1164 Sofia, Bulgaria

**Keywords:** Multivariate statistics, Greenness assessment, Green chemistry, Benzo[*a*]pyrene, Chemometrics

## Abstract

This study presents an application of the Hasse diagram technique (HDT) as the assessment tool to select the most appropriate analytical procedures according to their greenness or the best analytical performance. The dataset consists of analytical procedures for benzo[*a*]pyrene determination in sediment samples, which were described by 11 variables concerning their greenness and analytical performance. Two analyses with the HDT were performed—the first one with metrological variables and the second one with “green” variables as input data. Both HDT analyses ranked different analytical procedures as the most valuable, suggesting that green analytical chemistry is not in accordance with metrology when benzo[*a*]pyrene in sediment samples is determined. The HDT can be used as a good decision support tool to choose the proper analytical procedure concerning green analytical chemistry principles and analytical performance merits.

**Electronic supplementary material:**

The online version of this article (doi:10.1007/s00216-016-9473-4) contains supplementary material, which is available to authorized users.

## Introduction

Polycyclic aromatic hydrocarbons (PAHs) include a wide class of hazardous organic compounds that consist of two or more benzene rings in linear, angular, or cluster arrangements [1]. Because of the short-term and long-term toxicity of PAHs and their persistence or ability to bioaccummulate in the environment, these compounds have become the focus of much attention in recent years [2]. Sixteen PAHs known as priority pollutants are on the lists established by agencies responsible for environmental resource management in Europe and the USA [3]. Some species of PAHs have been classified as either possible or probable human carcinogens and mutagens by experts from the International Agency for Research on Cancer [4]. PAHs are metabolized to dihydrodiols by hydrocarbon hydroxylases present in the liver. Benzo[*a*]pyrene as a potent carcinogenic species has been chosen by certain agencies and organizations as an indicator of total PAHs [5]. The toxicities of other PAHs are often compared with the toxicity of benzo[*a*]pyrene.

PAHs are ubiquitous organic micropollutants [6]. They are mainly formed during the incomplete combustion of natural and organic materials (e.g., coal, fossil fuels, tobacco, or smoked meat) [7, 8]. Distribution of PAH mixtures into the environment is also caused by industrial emissions (e.g., coal gasification, aluminum production, steel founding) or traffic exhausts. Atmospheric PAHs, as particulates or gases, can be deposited on the surface of water. Because of their high lipophilicity and low biodegradability, PAHs can be absorbed by particulate materials rich in organic matter and accumulate at the bottom of lakes or rivers [9]. Hazardous compounds present in sediments can be a potential danger to aquatic ecosystems or drinking water. Understanding of the impact of particular emission sources on the different compartments of the aquatic environment is crucial for proper risk assessment and risk management. For this purpose reliable analytical procedures are required, and many have been proposed.

The quantitative determination of PAHs in sediments is a challenging task because of certain differences in the polarity, water solubility, and volatility of these compounds, their low concentration in samples, and the potential for the presence of matrix interferences. Thus, several methods have been described for the determination of PAHs, applying different extraction, purification, and detection methods. Chromatographic techniques, mainly gas chromatography [10] and high-performance liquid chromatography [11] have usually been applied for the determination of PAHs in sediments. Chromatographic analyses require sample pretreatment. For complex matrices such as sediment samples, Soxhlet extraction or liquid–solid extraction are the methods recommended by the US Environmental Protection Agency and the US National Oceanic and Atmospheric Administration [12, 13]. However, these extraction methods are highly time-consuming and labor intensive [14]. Moreover, their introduction in routine analysis is related to the use of a large volume of toxic solvents, which in consequence can result in long exposure of laboratory personnel. Increase in the energy costs and the amount of waste constitutes another very important aspect in analytical processes. Considering all these disadvantages, the sample preparation has proven to be the most significant stage in the whole analytical procedure and the most polluting in most procedures applying traditional methods [15].

The main goal of green analytical chemistry [16], which emerged from green chemistry, is to reach a compromise between the quality of the results and improving the environmental friendliness of analytical methods [17]. Because of special analytical criteria that need to be considered, such as limits of detection (LODs), limits of quantitation, precision, and accuracy, in some cases it is impossible to meet certain green analytical chemistry requirements in analytical methods [18]. In this situation, some approaches to make analytical procedures greener should be included.

Different methods can be used for determination of a single analyte. There are a few assessment methods allowing the evaluation of the “greenness” of each analytical procedure; use of the National Environmental Methods Index (NEMI) is one of them [19]. The greenness of an analytical method is assessed by use of the greenness profile symbol, which is divided into four fields, each describing different aspects of the environmental impact of the method; that is, (1) persistence, bioaccumulation potential, and toxicity, (2) hazardousness, (3) corrosiveness, and (4) waste. The procedure is considered green and the pictogram field is filled green if the following requirements are met: none of the chemicals used during the procedure are listed on the persistence, bioaccumulation potential, and toxicity chemicals lists or is present on the K, F, P, or U hazardous lists (F list for nonspecific source waste, K list for source-specific waste, and P and U lists for discarded commercial chemicals), the pH of the sample is within the 2–12 range, and less than 50 g of waste is produced.

The second very important tool used for assessing the greenness of analytical procedures is the analytical Eco-Scale [20]. The result of analysis with Eco-Scale is expressed as a number lower than 100. Penalty points which are allotted for the amount and type of chemical reagent, energy consumption, analyst occupational hazard, solid waste generated, and the way solid waste is treated (or not treated) are subtracted from the initial value of 100.

Benzo[*a*]pyrene was chosen as the representative of the group of PAHs to be analyzed in this study. The aim of the study is to obtain information about similarities or dissimilarities between analytical procedures applied to the determination of benzo[*a*]pyrene in sediments by use of multivariate statistical techniques, which will be significant in selecting the “latent” factors determining the greenness of analytical procedures studied. We investigate the possibility of applying the Hasse diagram technique (HDT) as a metrics tool for green analytical chemistry taking into account the analytical performance of the analytical procedures. The HDT allows for partial ordering of multivariate sets. The results obtained from analysis of Eco-Scale and NEMI as methods for the assessment of analytical method greenness will be also discussed.

## Materials and methods

### Input data for the analysis

All the analytical procedures included in the HDT analysis were developed for the determination of benzo[*a*]pyrene in sediment samples. The input data for the multivariate statistical analysis were collected from published articles. The publishers’ databases, including American Chemical Society, Taylor and Francis Online database, Royal Society of Chemistry Journals online database, ScienceDirect, SpringerLink, and Wiley Online Library database, were searched for the appropriate analytical procedures. No time frame or any limitations concerning the analytical techniques were applied. The procedure was included only if all the required information could be extracted from the article; otherwise the analytical procedure was rejected from further analysis. The analytical procedures extracted for the statistical analyses are presented in Table 1.

**Table 1:** Analytical procedures used as input data for multivariate analysis of National Environmental Methods Index (*NEMI*; in the form of the number of green fields) and Eco-Scale results

No.	Analytical technique	NEMI score	Eco-Scale score	Reference
1	Ultrasonic solid–liquid extraction–thin-layer chromatography–high-performance liquid chromatography–diode array detection/ultraviolet detection	2	72	[21]
2	Ultrasonic solid–liquid extraction–high-performance liquid chromatography–programmable fluorescence detection	1	51	[22]
3	Ultrasonic solid–liquid extraction–high-performance liquid chromatography–programmable fluorescence detection	2	62	[23]
4	Ultrasonic micellar extraction–high performance liquid chromatography–ultraviolet detection	4	77	[24]
5	Vortex-assisted extraction–dispersive liquid–liquid microextraction–high-performance liquid chromatography–fluorescence detection	2	81	[25]
6	Ultrasonic-assisted extraction–matrix solid-phase dispersion–high-performance liquid chromatography–ultraviolet detection	2	89	[26]
7	Focused ultrasonic solid–liquid extraction–high-performance liquid chromatography–fluorescence detection	3	86	[11]
8	Micro focused ultrasonic solid–liquid extraction–high-performance liquid chromatography–fluorescence detection	2	83	[27]
9	Miniaturized homogenous liquid–liquid extraction–high-performance liquid chromatography–fluorescence detection	2	51	[28]
10	Microwave-assisted extraction–solid-phase extraction–liquid chromatography–photodiode array detection–mass spectrometry	2	59	[29]
11	In situ microwave-assisted extraction–high-performance liquid chromatography–photodiode array detection	2	81	[30]
12	Microwave-assisted extraction–high-performance liquid chromatography–ultraviolet detection	3	78	[24]
13	Microwave-assisted extraction–high performance liquid chromatography–fluorescence detection	2	61	[31]
14	Microwave-assisted extraction–gas chromatography–mass spectrometry	2	60	[32]
15	Microwave-assisted extraction–2-dimensional gas chromatography–time-of-flight mass spectrometry	2	56	[33]
16	Microwave-assisted extraction–gas chromatography–mass spectrometry	2	53	[34]
17	Soxhlet extraction–gas chromatography–mass spectrometry	1	63	
18	Microwave-assisted extraction–gas chromatography–mass spectrometry	2	74	[35]
19	Soxhlet extraction–gas chromatography–mass spectrometry	2	68	
20	Accelerated solvent extraction–supercritical fluid extraction–gas chromatography–mass spectrometry	2	83	[36]
21	Focused ultrasonic solid–liquid extraction–gas chromatography–mass spectrometry	2	65	[37]
22	Microwave-assisted solid-phase extraction–gas chromatography–mass spectrometry	2	75	[38]
23	Microwave-assisted headspace solid-phase microextraction–gas chromatography–tandem mass spectrometry	4	92	[39]
24	Microwave-assisted micellar solid-phase microextraction–gas chromatography–mass spectrometry	3	93	[40]
25	Pressurized hot water extraction–solid-phase microextraction–gas chromatography–mass spectrometry	4	90	[41]
26	Pressurized liquid extraction–stir bar sorptive extraction–thermal desorption–gas chromatography–triple quadrupole mass spectrometry	2	77	[42]
27	Pressurized liquid extraction–solid-phase extraction–gas chromatography–mass spectrometry	2	68	[43]
28	Pressurized liquid extraction–gas chromatography–mass spectrometry	2	69	[44]
29	Pressurized liquid extraction–large-volume injection–gas chromatography–mass spectrometry	2	86	[45]
30	Programmed temperature vaporization–gas chromatography–mass spectrometry	2	60	[46]
31	Solid-phase extraction–gas chromatography–mass spectrometry/selected ion storage	2	84	[9]
32	Solid-phase extraction–gas chromatography–quadrupole ion trap mass spectrometry	2	81	[47]
33	Solid–liquid extraction–gas chromatography–tandem mass spectrometry–pseudo multiple reaction monitoring	3	90	[48]
34	Ultrasonic solid–liquid extraction–solid-phase extraction–gas chromatography–mass spectrometry	2	68	[49]
35	Ultrasonic solid–liquid extraction–gas chromatography–mass spectrometry	2	62	[50]
36	Ultrasonic solid–liquid extraction–gas chromatography–mass spectrometry	2	53	[10]
37	Ultrasonic solid–liquid extraction–gas chromatography–mass spectrometry	2	74	[4]
38	Ultrasonic solid–liquid extraction–stir bar sorptive extraction–thermal desorption–gas chromatography–mass spectrometry	2	55	[51]
39	Ultrasonic solid–liquid extraction–gas chromatography–electron ionization tandem mass spectrometry	2	60	[52]
40	Ultrasonic solid–liquid extraction–thin-layer chromatography–gas chromatography–ion trap mass spectrometry	2	60	[21]
41	Online dynamic microwave-assisted extraction–solid-phase extraction–gas chromatography–mass spectrometry	2	67	[53]

All the procedures presented in Table 1 are described by the variables in such a way that 26 of the procedures were described by both metrological and greenness data and all 41 procedures were characterized by greenness variables only. The variables included in the chemometric analyses are presented and explained in Table 2. Completeness of data availability was the requirement to include the procedure in the chemometric analyses.

**Table 2:** The variables considered during multivariate data analysis

Variable	Units	Remarks
Limit of detection	ng g^-1^	–
Precision	%	–
Recovery	%	–
Amount of sample	g	The sample mass subjected to the analysis
Number of other analytes determined	Unitless	Number of analytes, other than benzo[*a*]pyrene, determined in a single analytical run
Amount of organic solvent	mL	The total amount of all organic solvents used in the analytical protocol
Amount of organic solvent × hazard		The total amount of each organic solvent used in the analytical protocol multiplied by its hazard. In the case of a warning pictogram, the multiplier is 1; in the case of a danger pictogram, the multiplier is 2
Amount of solid waste	g	The total mass of all waste generated during analysis with the analytical protocol
Time	h	Estimated total time to analyze the sample
NEMI score	Unitless (range 0–4)	NEMI score calculated for each analytical method
Eco-Scale score	Unitless (range 0–100)	Eco-Scale score calculated for each analytical method

*NEMI* National Environmental Methods Index

For detailed information on the input data, see the electronic supplementary material.

### Principal component analysis

Principal component analysis is a well-known method widely applied in analytical chemistry, so it will not be described. All principal component analysis calculations were performed in PLS Toolbox for MATLAB.

### Hasse diagram

The HDT is applied to visualize relations of partial order between objects (in this case analytical procedures) described by a certain number of variables. The HDT is well described in the literature [54–56], and only a brief description will be given here.

In the HDT the ranking of analytical procedures is done with respect to variables (i.e., metrological or environmental), which is called the “information basis” (IB). The processed data matrix **Q** (*N* × *R*) contains *N* procedures and *R* variables. Entry y_*ir*_ of **Q** is the numerical value of the *r*th variable for the *i*th procedure. Two objects *s* and *t* are comparable if

$$ s\le t\iff y(s)\le y(t), $$

*y*(*s*) ≤ *y*(*t*) ⇔ *y*_*r*_(*s*) ≤ *y*_*r*_(*t*) for all *y*_*r*_ ∈ IB.

If there is at least one *y*_*r*_ for which *y*_*r*_(*s*) > *y*_*r*_(*t*), then the objects s and t are incomparable. A partially ordered set can be easily developed by a covering relation matrix which collects relations between each pair of procedures.

The relations stored in the covering relation matrix can be visualized by a Hasse diagram. For construction of the Hasse diagram, a uniform orientation of the variables should be secured; that is, high variable values correspond to “bad” procedures and low values correspond to “good” procedures [57]. In the present study the procedures near the upper part of the Hasse diagram indicate procedures that are characterized by poorer analytical (Fig. 3) or environmental (Fig. 2) performances.

The objects in a Hasse diagram that are not covered by other objects are called “maximal objects.” Objects which do not cover other objects are called “minimal objects.” In some diagrams there are also isolated objects which can be considered as maximal and minimal objects at the same time (objects 24 and 29 in Fig. 2). A chain is a set of comparable objects; therefore levels can be defined as the longest chain within the diagram. An antichain is a set of mutually incomparable objects, located at one and the same level. The height of the diagram is the longest chain, and the longest antichain is its width.

The number of incomparable elements in the Hasse diagram may obviously constitute a limitation in the attempt to rank the objects (analytical procedures) according to their variables. To a certain extent this problem can be remedied through the application of the so-called linear extensions of the partial order ranking. A linear extension is a total order, where all covering relations of the partially ordered set are reproduced [58]. Because of the incomparable elements in the partial order ranking, a number of possible linear extensions correspond to one partial order. However, the number of linear extensions goes with *N*! for a partially ordered set with *N* objects, and only for cases with a relatively low number of objects (fewer than 25) is an exact method for calculating average ranks available [58]. In the present study, averaged ranks of analytical procedures were calculated by a linear extension set obtained by the Bubley–Dyer algorithm [59].

All calculations concerning the HDT were performed with PyHasse [60] which is available on request from the developer, R. Bruggemann (e-mail: brg_home@web.de).

## Results and discussion

**Fig. 1:**
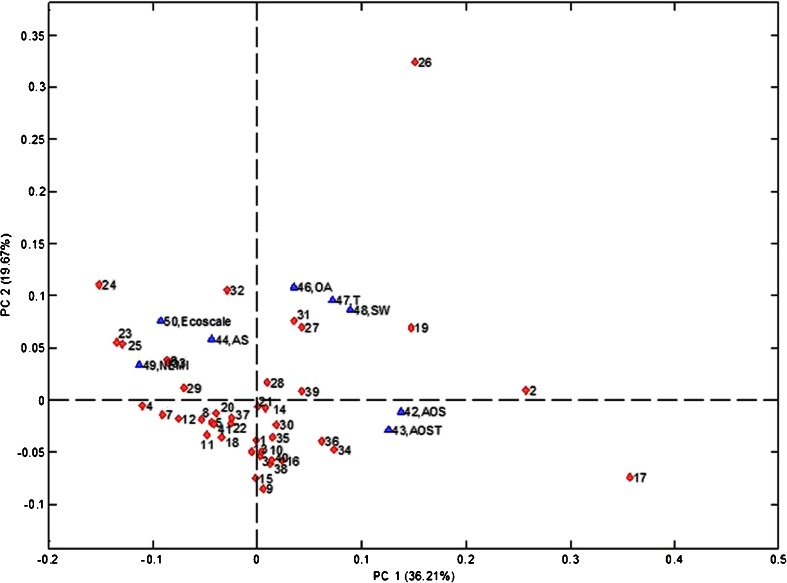
The results of principal component (*PC*) analysis for the “environmental” variables from Table 2. *AOS* amount of organic solvent, *AOST* amount of organic solvent × hazard, *AS* amount of sample, *NEMI* National Environmental Methods Index, *OA* number of other analytes determined, *SW* amount of solid waste, *T* time

To obtain general information about the dataset structure, PCA was performed; the results are presented in Fig. 1. The LOD, recovery, and precision were not reported in some of source articles listed in Table 1; therefore these variables were not included in the initial assessment.

The results show that the most of the analytical procedures form one group but there are some outliers. For example procedures 2, 17, and 26, are definitely outliers; they might be potentially performing both much better or much worse according to the variables presented.

The other interesting information is the correlation among the variables. The assessment results obtained by means of NEMI and Eco-Scale are well correlated with each other and they correlate well with the amount of sample. Similarly, the amount of solvent and the hazard-weighted amount of solvent are well correlated, which seems to be obvious. The third group of variables is formed by the analysis time, the amount of solid waste generated during analysis, and the number of other analytes that are determined during a single analysis. This can be interpreted in a way that multianalyte procedures require more time to perform quantitative analyte extraction, which also requires more solid reagent inputs (without recalculation to obtain “per analyte” inputs). All these findings are in agreement with the results of previous studies with the self-organizing maps approach for aldrin determination [61] and benzene and phenol determination [62] in water samples.

**Fig. 2:**
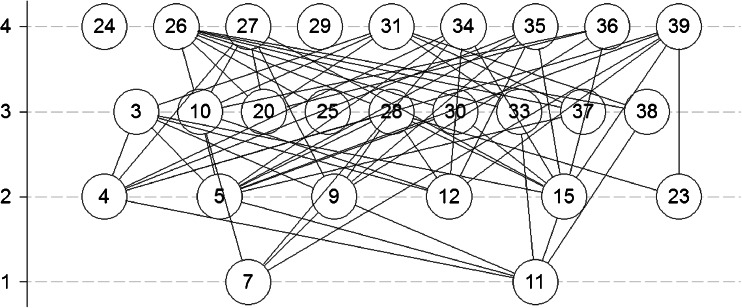
Hasse diagram obtained after analysis with the “environmental” variables described in Table 2

The assessment with the HDT was performed for a limited dataset because of low data availability. Some of the variable values (especially recoveries) were not reported in the source articles. Ranking with the HDT for the environmental variables included is shown in Fig. 2.

The results show that procedures 7 and 11 (see Table 1) are ranked as the most environmentally friendly. Both of these methods are characterized by low consumption of solvents which are of low toxicity, require a low sample mass, and produce no waste. The methods present in the maximal objects layer can be treated as the most environmentally problematic. They are procedures based on pressurized liquid extraction and ultrasonic solid–liquid extraction. This suggests that procedures based on ultrasonic solid–liquid extraction and pressurized liquid extraction are less green than procedures based on microwave-assisted extraction, which are present in the second and third layers of the Hasse diagram. There are also two procedures that are isolated objects. Procedures 24 and 29 (see Table 1) are incomparable with the other procedures. This is because they are characterized by very low values of certain variables and high values of other variables. In this case both procedures consume no or virtually no solvents and produce no waste. On the other hand, the analyses with these procedures are time consuming and require considerable amounts of samples. Both procedures cannot be disregarded as green alternatives and should be treated as a separate group in further considerations.

To check the assessment with the HDT, the results were compared with the results of NEMI and Eco-Scale assessment, which are established assessment procedures and might be treated as reference assessment methods. Comparison with the NEMI assessment (data not shown) results does not show any dependence as most of the methods are scored with two green fields (73 % of the methods). The HDT as a green analytical chemistry metrics assessment technique (with well-selected variables as input data) has better resolution power than NEMI labeling. Comparison of the HDT results with the Eco-Scale assessment scores is more informative. The mean Eco-Scale scores for diagram levels 1, 2, 3, and 4 are 83.5, 72.5, 71.3 and 67.4 respectively. The mean Eco-Scale score for two incomparable procedures is 89.5, which confirms the fact that they also should be treated as green analytical procedures.

**Fig. 3:**
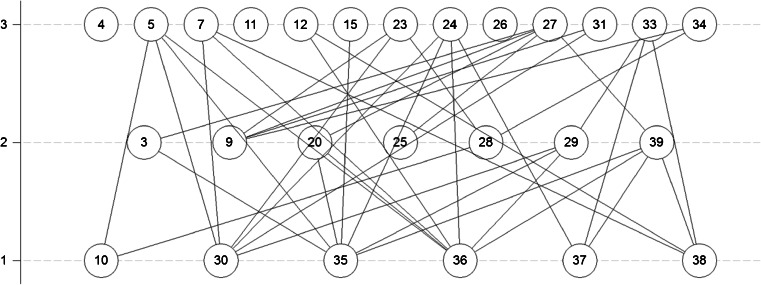
Hasse diagram obtained after analysis with the “metrological” variables described in Table 2

Figure 3 presents the HDT assessment results with metrology-related variables as input data. The most beneficial procedures are located at the lowest Hasse diagram level as these procedures are characterized by lower LODs and precision, and recoveries closer to 100 %. The best procedures in terms of analytical performance are those based on ultrasound-assisted solvent extraction, followed by gas chromatography–mass spectrometry. On the second level of the Hasse diagram there are mainly methods based on gas chromatography–mass spectrometry based mainly with pressurized liquid extraction as the sample preparation technique. The three objects that are incomparable are objects 4, 11, and 26 (see Table 1). They are characterized by a very low LOD and bad precision or vice versa.

**Fig. 4:**
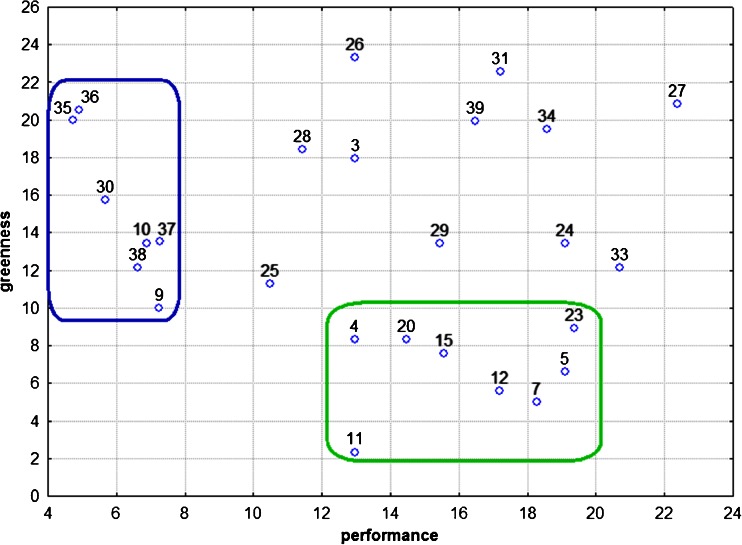
A bivariate plot of the averaged rank of analytical procedures based on metrological (performance) and environmental (greenness) variables

Linear extension analysis of the metrological and environmental performance of analytical procedures for benzo[*a*]pyrene determination can be very interesting. Figure 4 presents a graph with calculated average ranks according to metrological and environmental variables. The most important message conveyed by Fig. 4 is that there is no procedure that has both good metrological and good environmental performance. A group of procedures (in the green frame in Fig. 4) meet the green analytical chemistry criteria and another group o procedures (in the blue frame in Fig. 4) have good analytical performance. It is hard to choose the procedure that meets both criteria; certain trade-offs are needed. From Fig. 4 it can be easily seen that the procedures in the lower-left part of the diagram (4, 9, 11, 25 and 38) have best the compromise of analytical and environmental performances. It is very easy to eliminate the procedures that are characterized by low overall performance—they are located in the upper-right part of the diagram, such as 27, 31, 34, and 39. It can be concluded that depending on the requirements for the particular monitoring study (LOD, precision, etc.), the proper analytical procedure in the lower-left part of the graph can be chosen. It cannot be expected that the HDT will afford a “final decision” about the greenness of a large number of analytical procedures. By the present study we offer a new opportunity for the ranking o analytical procedures using the most specific variables related to the assessment of the greenness summarized as “performance” and “greenness” parameters. As seen in Fig. 4, quite good assessment is achieved, allowing the ranking of analytical procedures. In our opinion the results sufficiently reflect the goals of the study.

## Conclusions

The HDT can be used as a convenient tool to compare the performance of a set of analytical procedures. The HDT allowed us to choose the best analytical procedures for benzo[*a*]pyrene determination in sediment samples according to metrological parameters; they were mainly procedures based on ultrasound-assisted extraction. Introduction of variables based on environmental impact as input data for the HDT allowed us to rank the procedures according to their green character. Generally, the procedures based on microwave-assisted extraction were greener, although it is hard to make authoritative generalizations. The HDT was found to be a reliable green analytical chemistry assessment tool. The assessment results are in good agreement with the Eco-Scale assessment results. Another conclusion is that there are no procedures for benzo[*a*]pyrene determination in sediment samples that are characterized by both good analytical and good environmental performance.

## Electronic supplementary material

Below is the link to the electronic supplementary material.

ESM 1 (PDF 64 kb)
