# Supplementary material for: Hasse diagram as a green analytical metrics tool: ranking of methods for benzo[a]pyrene determination in sediments
Source: Anal Bioanal Chem. 2016 Apr 1;408:3833–41. doi: 10.1007/s00216-016-9473-4 (PMC4848339; doi:10.1007/s00216-016-9473-4)
Supplement: Supplementary file 1 — (PDF 64 kb) [file 216_2016_9473_MOESM1_ESM.pdf]

Analytical and Bioanalytical Chemistry

Electronic Supplementary Materials

Hasse diagram as a green analytical metrics tool: ranking of methods for benzo[a]pyrene determination in sediments

Paulina Bigus, Stefan Tsakovski, Vasil Simeonov, Jacek Namieśnik, Marek Tobiszewski

Table S1 Full input data to HDT analysis

No	Technique abbreviation	Analytical technique	LOD [ng/g]	RSD [%]	Recovery [%]	Amount of organic solvent [ml]	Amount of organic solvent * toxicity (hazard)	Amount of sample [g]	injection volume [ul]	other analytes (per analyte concept)	time [h]	soild waste (g)	NEMI score	Eco-scale score	Ref.
1	USLE-TLC-HPLC-DAD/UV	Ultrasonic solid-liquid extraction-thin-layer chromatography-high performance liquid chromatography – diode array detection-ultraviolet detection	-	8	78	124.94	501.76	10	20	11	2.9	0	2	72	[1]
2	USLE-HPLC-PFD	Ultrasonic solid-liquid extraction-high performance liquid chromatography-programmable fluorescence detection	-	3.05	93.7	487	1961	20	2000	14	1.9	60	1	51	[2]
3	USLE-HPLC-FLD	Ultrasonic solid-liquid extraction-high performance liquid chromatography-programmable fluorescence detection	1.12	12.1	108.73	72.9	383.4	5	20	14	1.75	0	2	62	[3]
4	UME-HPLC/UV	Ultrasonic micellar extraction-high performance liquid chromatography-ultraviolet detection	50	4.25	94.1	33.25	133	0.8	25	10	1.4	0	4	77	[4]
5	VAE-DLLME-HPLC-FLD	Vortex-assisted extraction- dispersive liquid-liquid microextraction-high performance liquid chromatography-fluorescence detection	2.6	4.9	74.9	25.44	101.52	0.2	10	14	0.82	0	2	81	[5]
6	UAE-MSPD-HPLC/UV	Ultrasonic assisted extraction-matrix-solid phase dispersion – high performance liquid chromatography – ultraviolet detection	-	-	-	1.5	6	100	1000	14	1	0.4	2	89	[6]
7	FUSLE-HPLC-FLD	Focused ultrasonic solid-liquid extraction-high performance liquid chromatography-fluorescence detection	9.3	3.9	79.2	29.80	119.2	1	20	6	0.7	0	3	86	[7]
8	μFUSLE-HPLC-FLD	Micro-focused ultrasonic solid-liquid extraction-high performance liquid chromatography-fluorescence detection	-	14	94	3.9	15.6	0.125	60	13	1.0	0	2	83	[8]
9	MHLLE-HPLC-FLD	Miniaturized homogenous liquid-liquid extraction-high performance liquid chromatography-fluorescence detection	0.003	7	87	43.525	196.15	2	20	8	1.1	0	2	51	[9]
10	MAE-SPE-LC-DAD-MS	Microwave-assisted extraction- solid phase extraction- liquid chromatography-photodiode array detection- mass spectrometry	0.0016	3.4	75.9	55.0	282.5	1	20	19	0.84	1	2	59	[10]
11	MAE-HPLC-DAD	*in-situ* Microwave-assisted extraction- high performance liquid chromatography-photodiode array detection	1000	3.4	102	18.10	72.4	0.1	200	8	0.82	0	2	81	[11]
12	MAE-HPLC/UV	Microwave-assisted extraction-high performance liquid chromatography-ultraviolet detection	40	3.4	84.4	33.25	133	0.8	25	11	0.8	0	3	78	[12]

13	MAE-HPLC-FLD	Microwave-assisted extraction-high performance liquid chromatography-fluorescence detection	-	-	-	33.85	140.8	0.3	5	15	0.9	2.65	2	61	[13]
14	MAE-GC-MS	Microwave-assisted extraction- gas chromatography-mass spectrometry	-	8.3	73.7	45.50	163.5	2	1	37	2	1	2	60	[14]
15	MAE-GC×GC-TOF-MS	Microwave-assisted extraction- gas chromatography-time-of-flight-mass spectrometry	31	7	92.2	36	227.2	0.5	1	9	1.2	0	2	56	[15]
16	MAE-GC-MS	Microwave-assisted extraction-gas chromatography-mass spectrometry	-	6.5	69	55	368	1	1	13	0.8	9	2	53	[16]
17	SOXE-GC-MS	Soxhlet extraction-gas chromatography-mass spectrometry	-	5.9	77	770	6128	1	1	13	16.6	9	1	63	
18	MAE-GC-MS	Microwave-assisted extraction-gas chromatography-mass spectrometry	-	5.8	95	30	30	1	1	12	1.2	0	2	74	[17]
19	SOXE-GC-MS	Soxhlet extraction-gas chromatography-mass spectrometry	-	4.5	95.3	500	500	1	1	12	49.03	0	2	68	
20	ASE-SFE-GC-MS	Accelerated solvent extraction-supercritical fluid extraction-gas chromatography- mass spectrometry	2.28	11.2	88	60.00	60	5	2	14	1.0	2	2	83	[18]
21	FUSLE-GC-MS	Focused ultrasonic solid-liquid extraction-gas chromatography- mass spectrometry	0.16	12	-	23.20	385.12	1	2	35	0.6	2	2	65	[19]
22	MA-SPE-GC-MS	Microwave-assisted-solid-phase extraction-gas chromatography – mass spectrometry	-	6.4	117.7	48	193	5	1	17	2	0	2	75	[20]
23	MA-HS-SPME-GC-MS/MS	Microwave-assisted-headspace solid-phase microextraction-gas chromatography-tandem mass spectrometry	0.5	9	22	0.90	3.6	1	TD	26	1.9	0	4	92	[21]
24	MA-MSPME-GC-MS	Microwave-assisted-micellar solid-phase microextraction-gas chromatography-mass spectrometry	4.8	5.1	82.8	0.00	0	200	TD	15	3	0	3	93	[22]
25	PHWE-SPME-GC-MS	Pressurized hot water extraction-solid-phase microextraction-gas chromatography- mass spectrometry	9.0	9.8	97.7	2	12	0.5	TD	26	2.73	0	4	90	[23]
26	PLE-SBSE-TD-GC-MS/MS QqQ	Pressurized liquid extraction- stir-bar sorptive extraction- thermal desorption- gas chromatography-triple quadrupole mass spectrometry	0.002	31	110	65.0	450	10	TD	86	37	60	2	77	[24]
27	PLE-SPE-GC-MS	Pressurized liquid extraction- solid phase extraction- gas chromatography- mass spectrometry	24.6	22.2	84.7	208.0	321	25	1	63	1.62	5	2	68	[25]
28	PLE-GC-MS	Pressurized liquid extraction-gas chromatography-mass spectrometry	0.3	4.3	72.6	50.0	330	5	1	33	2	10	2	69	[26]
29	PLE-LVI-GC-MS	Pressurized liquid extraction-large volume injection-gas chromatography-mass spectrometry	3	5	80	0.1	0.6	50	50	15	0.7	0	2	86	[27]

30	PTV-GC-MS	Programmed temperature vaporization- gas chromatography- mass spectrometry	0.44	2.5	84.1	60.3	378	0.5	25	25	2.9	5	2	60	[28]
31	SPE-GC-QIT-MS/SIS	Solid-phase extraction- gas chromatography- mass spectrometry/selected ion storage	0.7	7.1	121	117	516	10	1	19	16.4	22.2	2	84	[29]
32	SPE-GC-MS	Solid-phase extraction- gas chromatography- quadrupole ion trap mass spectrometry	-	5.5	-	19.5	48	100	2	22	24.9	1	2	81	[30]
33	SLE-GC-MS/MS-PMRM	Solid-liquid extraction-gas chromatography-tandem mass spectrometry-pseudo multiple reaction monitoring	7.5	5	80	20	80	10	1	17	1.35	8	3	90	[31]
34	USLE-SPE-GC-MS	Ultrasonic solid-liquid extraction-solid-phase extraction- gas chromatography- mass spectrometry	0.4	10	63	270.25	1700.63	1	1	15	1.84	5	2	68	[32]
35	USLE-GC-MS	Ultrasonic solid-liquid extraction- gas chromatography- mass spectrometry	1.0	4.6	96.7	100.2	601.2	15	1	22	1.7	0	2	62	[33]
36	USLE-GC-MS	Ultrasonic solid-liquid extraction- gas chromatography- mass spectrometry	2	2.4	111.9	170.025	905	5	25	28	1.3	5	2	53	[34]
37	USLE-GC-MS	Ultrasonic solid-liquid extraction-gas chromatography-mass spectrometry	4.6	4	92	27	162.1	10	1	15	1.4	5.2	2	74	[35]
38	USLE-SBSE-TD-GC-MS	Ultrasonic solid-liquid extraction-stir bar sorptive extraction-thermal desorption-gas chromatography-mass spectrometry	5.23	2	88.3	46	304	0.2	TD	11	6	0	2	55	[36]
39	USLE-GC-EI-MS/MS	Ultrasonic solid-liquid extraction- gas chromatography- electron ionization- tandem mass spectrometry	23	6	85	92.5	950	1	500	50	1.9	0	2	60	[37]
40	USLE-TLC-GC-MS/IT	Ultrasonic solid-liquid extraction-thin-layer chromatography – gas chromatography – mass spectrometry/ion trap	-	14	40	110.5	444	10	1	11	2.8	0.5	2	60	[1]
41	On-line-DMAE-SPE-GC-MS	On-line dynamic microwave-assisted extraction-solid-phase extraction-gas chromatography-mass spectrometry	-	1	91	8.9	54.6	60	1	12	0.9	0	2	67	[38]

[1] Filipkowska A. Lubecki L. Kowalewska G. Polycyclic aromatic hydrocarbon analysis in different matrices of the marine environment. Anal. Chim. Acta 2005;547:243–254.
[2] Williamson KS. Petty J.D. Huckins J.N. Lebo J.A. Kaiser E.M. HPLC-PFD determination of priority pollutant PAHs in water sediment and semipermeable membrane devices. Chemosphere 2003;49:703–715.
[3] Peng X. Yan G. Li X. Guo X. Zhou X. Wang Y. Optimization of Ultrasonic Extraction and Clean-up Protocol for the Determination of Polycyclic Aromatic Hydrocarbons in Marine Sediments by High-performance Liquid chromatography Coupled with Fluorescence Detection. J. Ocean Univer. China 2012;11:331–338.

[4] Pino V. Ayala JH. Afonso AM. Gonzalez V. Ultrasonic micellar extraction of polycyclic aromatic hydrocarbonś from marine sediments. Talanta 2001;54:15–23.

[5] Leng G. Lui G. Chen Y. Yin H. Dan D. Vortex-assisted extraction combined with dispersive liquid-liquid microextraction for the determination of polycyclic aromatic hydrocarbons in sediment by high performance liquid chromatography, J. Sep. Sci. 2012;35:2796–2804.

[6] Moliner-Martinez Y. Gonzalez-Fuenzalida R.A. Herraez-Hernandez R. Campins-Falco P. Verdu-Andres J. Cleaning sorbents used in matrix solid-phase dispersion with sonification: Applcation to the estimation of polycyclic aromatic hydrocarbons at ng/g levels in marine sediments, J. Chromatogr. A 2012;1263:43–50.

[7] Pino V. Anderson J.I. Ayala J.H. Gonzalez V. Afonso A.M. The ionic liquid 1-hexadecyl-3-methylimidazolium bromide as novel extracting system for polycyclic aromtic hydrocarbons contained in sediments using focused microwave-assisted extraction, J. Chromatogr. A 2008;1182:145–152.

[8] Capelo J.L. Galesio M.M. Felisberto G.M. Vaz C. Pessoa J.C. Micro-focused ultrasonic solid–liquid extraction (FUSLE) combined with HPLC and fluorescence detection for PAHs determination in sediments: optimization and linking with the analytical minimalism concept. Talanta 2005;66:1272–1280.

[9] Shampsipur M. Hassan J. A novel miniaturized homoggenous liquid-liquid solvent extraction-high performance liquid chromatographic-fluorescence method for determination of ultra traces of polycyclic aromatic hydroocarbons in sediments samples, J. Chromatogr. A 1217;2010:4877–4882.

[10] Gonazlo-Pinuela C. Alonso-Salces R.M. Andres A. Ortiz I. Viguri J.R. Validated analytical strategry for the determination of polycyclic aromatic compounds in marine sediments by liquid chromatography coupled with diode-array detection and mass spectrometry, J. Chromatogr. A 2006;1129:189–200.

[11] Delgado B. Pino V. Anderson J.L. Ayala J.H. Afonso A.M. Gonzalez V. An in-situ extraction-preconcentration method using ionic liquid-based surfactants for the determination of organic contaminants contained in marine sediments, Talanta 2012;99:972–983.

[12] Pino V. Ayala J.H. Afonso A.M. Gonzalez V. Ultrasonic micellar extraction of polycyclic aromatic hydrocarbons from marine sediments, Talanta 2000;54:15–23.

[13] Denis E.H. Toney J.L. Tarozo R. Anderson R.S. Roach L.D. Huang Y. Polycyclic aromatic hydrocarbons (PAHs) in lake sediments record historic fire events: Validations using HPLC-fluorescence detection. Org. Geochem. 2012;45:7–17.

[14] Thompson S. Budzinski H. LeMenach K. Letellier M. Garrigues P. Maulti-residue analysis of polycyclic aromatic hydrocarbons, polychlorobiphenyls, and organochlorine pesticides in marine sediments, Anal. Bioanal. Chem. 2002;372:196–204.

[15] Pena-Abaurrea M. Ye F. Blasco J. Ramos L. Evaluation of comprehensive two-dimensional gas chromatography-time-of-flight-mass spectrometry for the analytes of polycyclic hydrocarbons in sediments, J. Chromatogr. A 2012;1256:222–231.

[16] Shu Y.Y. Lao R.C. Chiu C.H. Turle R. Analysis of polycyclic aromatic hydrocarbons in sediment reference materials by microwave-assisted extraction, Chemosphere 2000;41:1709–1716.

[17] Budziński H. Letellier M. Garrigues P. Le Menach K. Optimisation of the microwave-assisted extraction in open cell of polycyclic aromatic hydrocarbons from soils and sediments. Study of moisture effect, J. Chromatogr. A 1999;837:187–200.
[18] Notar M. Leskovsek H. Determination of polycyclic aromatic hydrocarbons in marine sediments using a new ASE-SFE extraction technigue, Fresenius J. Anal. Chem. 2000;366:846–850.
[19] Errekatxo A. Prieto A. Zuloaga O. Usobiaga A. Etxebarria N. Fernandez L.A. Simultaneous extraction of several persistent organic pollutants in sediments using focused ultrasonic soild-liquid extraction. Anal. Bioanal. Chem. 2008;392:1471–1478.
[20] Itoh N. Numata M. Yarita T. Alkaline extraction in combination with microwave-assisted extraction followed by solid-phase extraction treatment for polycyclic aromatic hydrocarbons in sediment sample, Anal. Chim. Acta 2008;615:47–53.
[21] Herbert P. Silva A.L. Joao M.J. Santos L. Alves A. Determination of semi-volatile priority pollutants in landfill leachates and sediments using microwave-assisted headspace solid-phase microextraction, Anal. Bioanal. Chem. 2006;386:324–331.
[22] Pino V. Ayala J.H. Afonso A.M. Gonzalez V. Micellar microwave-assisted extraction combined with soild-phase microextraction for the determination of polycyclic aromatic hydrocarbons in certified marine sediment, Anal. Chim. Acta 2003;477:81–91.
[23] Fernandez-Gonalez V. Concha-Grana E. Muniategui-Lorenzo S. Lopez-Mahia P. Prada-Rodriquez D. Pressurized hot water extraction coupled to solid-phase microextraction-gas chromatography-mass spectrometry for analysis of polycyclic aromatic hydrocarbons in sediments. J. Chromatogr. A 2008b;1196-1197:65–72.
[24] Camino-Sanchez F.J. Zafra-Gomez A. Perez-Trujillo J.P. Conde-Gonzalez J.E. Mrques J.C. Vilchez J.L. Validation of a GC-MS/MS method for simultaneous determination of 86 persistent organic pollutants in marine sediments by pressurized liquid extraction followed by stir bar sorptive extraction, Chemosphere 2011;84:869–881.
[25] Burkhardt M.R. Re R. Vello R.C. Smith S.G. Zaugg S.D. Pressurized liquid extraction using water/isopropanol coupled with soild-phase extraction cleanup for industrial and anthropogenic waste-indicator compounds in sediment, Anal. Chim. Acta 2005;534:89–100.
[26] Choi M. Kim Y-J. Lee I.S. Choi H-G. Development of a one-step integrated pressurized liquid extraction and cleanup method for determining polycyclic aromatic hydrocarbons in marine sediments, J. Chromatogr. A 2014;1340:8–14.
[27] Ramos L. Vreuls J.J. Brinkman U.A.Th. Minaturised pressurised liquid extraction of polycyclic of aromatic hydrocarbons from soil and sediemnt with subsequent large-volume injection-gas chromatography, J. Chromatogr. A 2000;891:275–286.
[28] Fernandez-Gonalez V. Concha-Grana E. Muniategui-Lorenzo S. Lopez-Mahia P. Prada-Rodriquez D. A multivariate study of the programmed temperature vaporization injection-gas chromatography-mass spectrometric determination of polycyclic aromatic hydrocarbons. Application to marine sediments analysis, Talanta 2008;74:1096–1103.
[29] Leite N.F. Peralta-Zamora P. Grassi M.T. Multifactorial optimization approach for the determination for the determination of polycyclic aromatic hydrocarbons in river sediments by gas chromatography-quadrupole ion trap selected ion storage mass spectrometry, J.Chromatogr. A 2008;1192:273–281.

[30] Wolska L.Miniaturised analytical procedure of determining polycyclic aromatic hydrocarbons and polychlorinated biphenyls in bottom sediments, J. Chromatogr. A 2002;959:173–180.
[31]Shang D. Rapid and sensitive method for the determination of polycyclic aromatic hydrocarbons in soils using pseudo multiple reaction monitoring gas chromatography/tandem mass spectrometry, J. Chromatogr. A 2014;1334:113–125.
[32]Martinez E. Gros M. Lacorte S. Barcelo D. Simplified procedures for the analysis of polycyclic aromatic hydrocarbons in water, sediments and mussels, J. Chromatogr. A, 2004;1047:181–188.
[33]Banjoo D.R. Nelson P.K. Improved ultrasonic extraction procedure for the determination of polycyclic aromatic hydrocarbons in sediments, J. Chromatogr. A, 2005;1066:9–18.
[34] Planas C. Puig A. Rivera J. Caixach J. Analysis of alkyl and 2-6-ringed polycyclic aromatic hydrocarbons by isotope dilution gas chromatography/mass spectrometry. Quality assurance and determination in Spanish river sediments, J. Chromatogr. A 2006;1113:220–230.
[35] Santos MDR. Cerqueira MRF. de Oliveira MCL. Matos RC. Matos MAC. Box–Behnken design applied to ultrasoundassisted extraction for the determination of polycyclic aromatic hydrocarbons in river sediments by gas chromatography/mass spectrometry. Anal. Methods 2014;6:1650–1656.
[36] Yamaguchi Ch. Lee W.-Y. A cost effective, sensitive, and environmentally friendly sample preparation method for determination of polycyclic aromatic hydrocarbons in solid samples, J. Chromatogr. A 2010;1217:6816–6823.
[37] Sanchez-Avila J. Fernandez-Sanjuan M. Vincente J. Lacorte S. Development of a multi-residue method for the determination of organic micropollutants in water, sediment and mussels using gas chromatography-tandem mass spectrometry, J. Chromatogr. A 2011;1218:6799–6811.
[38] Ericsson M. Colmsjo A. Dynamic microwave-assisted extraction coupled on-line with solid-phase extraction: determination of polycyclic aromatic hydrocarbons in sediment and soil, J. Chromatogr. A 2002;964:11–20.
